# Surgical management of traumatic tricuspid regurgitation: a case report

**DOI:** 10.1093/ehjcr/ytae676

**Published:** 2024-12-19

**Authors:** Gianpiero Buttiglione, Daniel Höfer, Herbert Hangler, Nikolaos Bonaros

**Affiliations:** Department of University Cardiac Surgery, IRCCS Policlinico San Donato, Piazza Edmondo Malan, 2, 20097 Milan, Italy; Department of Cardiac Surgery, Medical University Innsbruck, Anichstrasse 35, A-6020 Innsbruck, Austria; Department of Cardiac Surgery, Medical University Innsbruck, Anichstrasse 35, A-6020 Innsbruck, Austria; Department of Cardiac Surgery, Medical University Innsbruck, Anichstrasse 35, A-6020 Innsbruck, Austria

**Keywords:** Trauma, Tricuspid valve injury, Blunt cardiac injury, Valve repair, Case report

## Abstract

**Background:**

Traumatic tricuspid valve regurgitation is a rare condition related to blunt chest trauma. In the early phase, the patients may remain asymptomatic. Progressive tricuspid regurgitation leads to the development of symptoms thereafter. Progressive right ventricular dysfunction aggravates symptoms, and the diagnosis is made by subsequent echocardiography at a later time. The treatment is usually surgical, especially in younger patients.

**Case summary:**

We describe a 30-year-old patient with traumatic tricuspid valve regurgitation after a motorcycle accident. No cardiac injury was detected at the moment of the collision, and the patient remained asymptomatic at the initial phase. Five years later, the patient was admitted to our hospital with symptoms of dyspnoea at exertion. Echocardiography demonstrated severe tricuspid valve regurgitation with right ventricle dilatation. Surgical tricuspid valve repair including ring annuloplasty and implantation of artificial chords via an endoscopic approach was performed. Surgery was complicated by impingement of the right coronary artery by one of the annuloplasty sutures, which was addressed by subsequent percutaneous coronary intervention.

**Discussion:**

Traumatic tricuspid valve regurgitation requires careful evaluation. Transthoracic echocardiography should be recommended to exclude post-traumatic tricuspid regurgitation after major blunt chest trauma. Early diagnosis is important to avoid right ventricular failure. First-line surgical treatment consists of tricuspid repair by means of ring annuloplasty and implantation of artificial chords.

**Learning points**

- Severe tricuspid regurgitation (TR) after trauma is a rare condition but should be excluded after major blunt trauma.
- The mechanism of traumatic TR is associated with papillary muscle rupture, rupture of primary chordae, leaflet laceration, or a combination of the above.
- Concomitant congenital anatomical conditions may aggravate post-traumatic tricuspid regurgitation.
- Avoiding tricuspid valve replacement is essential to improve prognosis.

## Introduction

Traumatic tricuspid valve regurgitation (TTVR) is the consequence of blunt chest trauma or penetrating injuries of the chest.^1^ The first report of traumatic injury of the tricuspid valve (TV) was described by William in 1829.^2^

The TTVR is a rare condition, and it involves 0.13% of the cases associated with common chest trauma.^1^ The most common underlying mechanism relates to rupture of primary chordae, the tip of the papillary muscle or leaflet laceration after blunt chest trauma or open chest injury, respectively.^3^

Commonly, the disease remains undiagnosed^4^ at the early stage, as bone fractures or bleeding of internal organs is prioritized. Severe TTVR leads to right ventricular dysfunction, which causes progressive symptoms such as fatigue, peripheral oedema, dyspnoea, and arrhythmias. The diagnosis is typically made by echocardiography in the presence of symptoms or an accidental detection of a systolic murmur at auscultation.

Treatment options depend on the severity of tricuspid regurgitation (TR) and the presence of clinical symptoms. According to the 2021 ESC/EACTS guidelines, surgery is recommended in symptomatic patients with isolated severe primary TR without severe right ventricle (RV) dysfunction.^5^

Early diagnosis and treatment are important to avoid right ventricular failure. Primary valve repair is the preferred surgical treatment to avoid oral anticoagulation, especially in younger patients. The TV replacement may be needed in the event of a non-reparable valve.

Our report describes the case of a patient with TTVR who was admitted to our hospital after a motorcycle accident 5 years earlier.

## Summary figure

**Figure:**
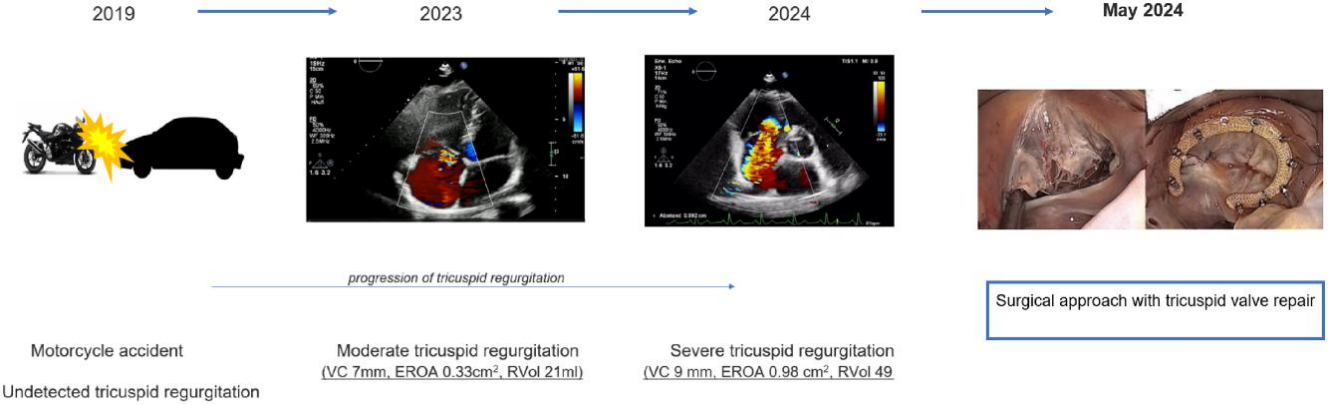


## Case presentation

A 30-year-old male presented at the cardiology outpatient clinic with symptoms of dyspnoea at exertion. The patient had a history of major motorcycle accident 5 years earlier. He survived a severe blunt thoracic trauma as well as complicated fractures of both lower extremities after a frontal collision of his motorcycle with a car. In addition, a blunt abdominal trauma causing a spleen rupture requiring splenectomy was noted. No cardiac injuries were suspected at this moment, and the patient was not submitted to any cardiac diagnostic workout.

Four years later, a systolic murmur was accidentally detected by his general practitioner while the patient remained asymptomatic. Subsequent transthoracic echocardiography (TTE) revealed moderate TR with a vena contracta (VC) of 7 mm, an effective regurgitant orifice area (EROA) of 0.33 cm^2^, and a regurgitant volume (RVol) of 21 mL due to an anterior tricuspid leaflet prolapse. In addition, there was a mild dilatation of the RV (mid-RV diameter: 30 mm) and right atrium (40 × 46 mm). No signs of RV dysfunction were detected (see Supplementary material).

**Figure 1:**
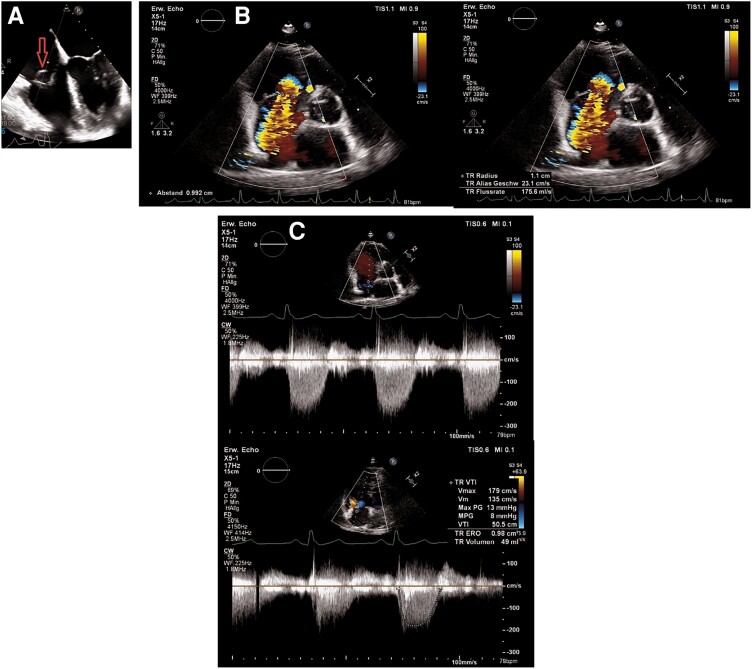
Preoperative transthoracic echocardiography demonstrating torrential tricuspid valve regurgitation. (*A*) Prolapse of the anterior tricuspid leaflet in apical four-chamber view (red arrow). (*B*) Quantitative assessment of tricuspid regurgitation using the proximal isovelocity surface area method. (*C*) The continuous wave Doppler signal of the regurgitant jet is truncated and triangular. Peak 1.7 m/s, effective regurgitant orifice area 0.98 cm^2^, and regurgitant volume 49 mL.

Seven months later, the patient was presented with fatigue and dyspnoea at exertion. Bilateral peripheral oedema with liver enlargement was found at physical examination. No additional pulmonary rales or signs of tachycardia were observed. A high pitched holosystolic murmur was identified at the left lower sternal border. Echocardiography demonstrated worsening of TV regurgitation from moderate to severe with a VC of 9 mm, a proximal isovelocity surface area radius of 11 mm, an EROA of 0.98 cm^2^, a RVol of 49 mL, and a severe dilatation of the RV (mid-RV diameter: 46 mm). Peak velocity of TR at continuous wave Doppler was 1.79 m/s (*Figure 1A–D*).

**Figure 2:**
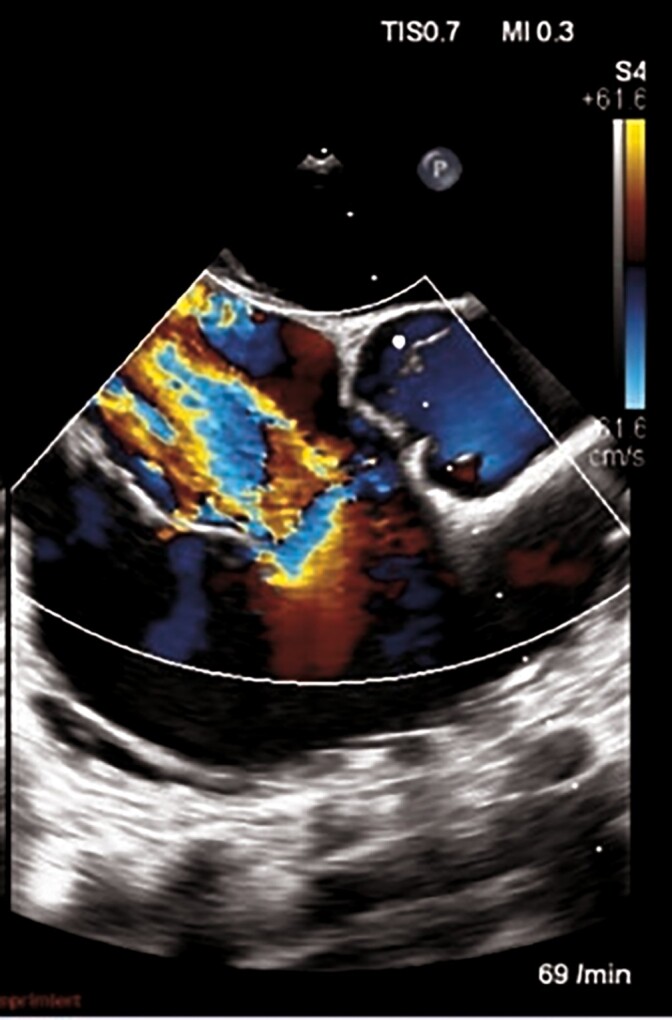
Intraoperative transesophageal echocardiography. Evidence of anterior leaflet tricuspid valve prolapse with rupture of primary chordae.

Surgical TV repair was indicated for the treatment of symptomatic severe TR according to the 2021 ESC/EACTS guidelines.^5^ The patient was deemed to be a good candidate for endoscopic tricuspid repair by the local heart team. Intraoperative transoesophageal echocardiogram (TOE) confirmed torrential TR with VC of 9 mm, EROA of 1.29 cm^2^, and a RVol of 62 mL (*Figure 2*).

The procedure was performed under femoro-femoral cardiopulmonary bypass via a 4 cm mini-thoracotomy above the right fourth intercostal space using three-dimensional endoscopy and long-shafted instruments.

**Figure 3:**
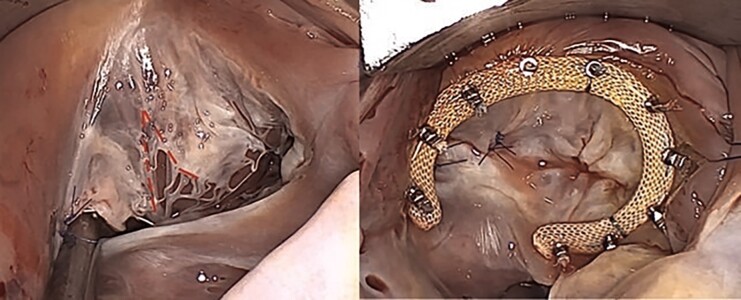
Intraoperative tricuspid valve. Cleft of the anterior leaflet (outlined by dashes, left panel). View of the tricuspid valve after repair (right panel).

Intraoperative findings revealed an anterior leaflet prolapse as a result of several ruptured primary chordae. The anterior leaflet was divided into an anterior and a posterior part by a cleft. The prolapsing segment of the anterior leaflet was resuspended using one polytetrafluoroethylene 4/0 artificial chord from the anterior papillary muscle. Three 4/0 polypropylene sutures were used to close the cleft. Finally, a 28 mm Physio Tricuspid Edwards annuloplasty ring was implanted with interrupted annuloplasty sutures (*Figure 3*). The patient was weaned off cardiopulmonary bypass without complications. The TOE demonstrated a competent valve with trivial regurgitation.

**Figure 4:**
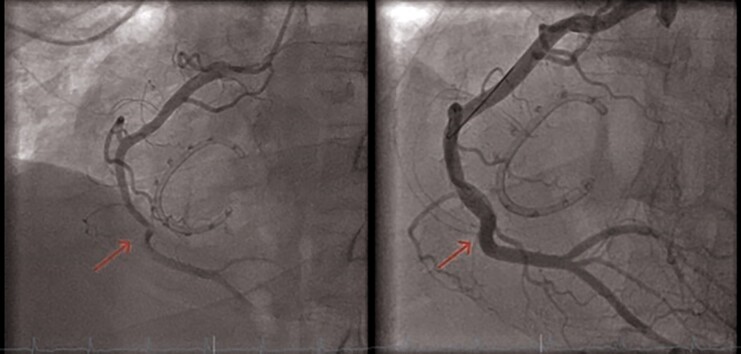
Right coronary artery treatment. Coronary angiography demonstrating impingement of the mid right coronary artery by annuloplasty sutures (red arrow, left panel). Coronary angiography after coronary stent implantation (red arrow, right panel).

**Figure 5:**
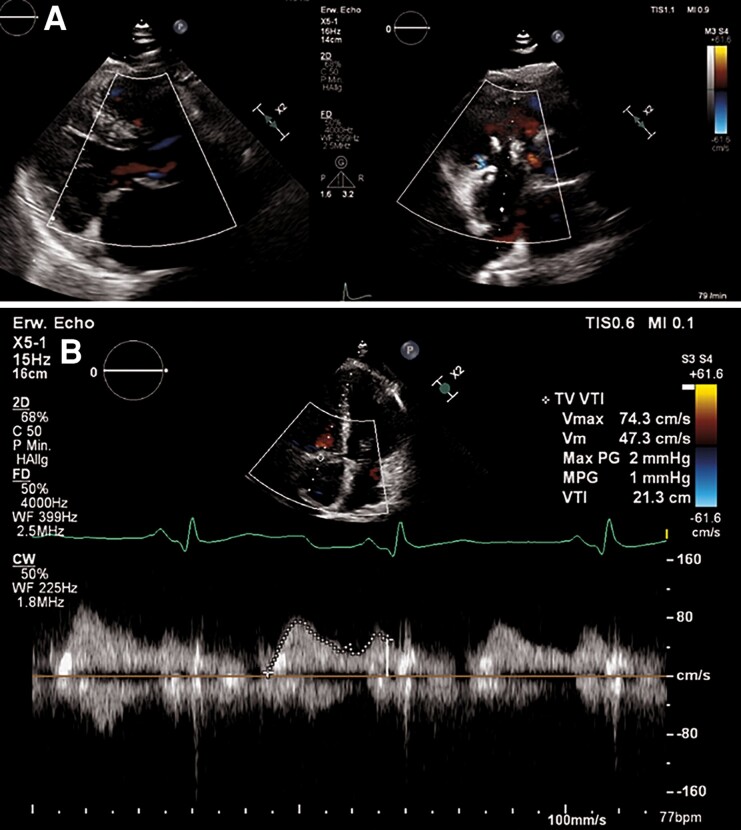
Pre-discharge transthoracic echocardiography with tricuspid valve repair. (*A*) Evidence of normal coaptation at the level of tricuspid valve annulus. (*B*) Trivial tricuspid valve regurgitation with mean pressure gradient 1 mmHg.

Unfortunately, on the first post-operative day, the patient developed ST-segment elevation detected at the posterior electrocardiogram-leads and high-sensitivity troponin T levels reached 3391 ng/L, the patient underwent urgent coronary angiography according to our internal protocols. The latter revealed an impingement of the mid-right coronary artery by one of the annuloplasty sutures. Percutaneous coronary intervention with a 4.0 × 22 mm drug eluting stent was performed (*Figure 4*). The remaining postoperative course was uneventful and there was only a trivial TR with mean pressure gradient of 1 mmHg at discharge echocardiography (*Figure 5A* and *B*).

## Discussion

Tricuspid regurgitation secondary to chest trauma is a rare condition requiring careful evaluation. The mechanism of TTVR is related to a sudden increase in RV pressure caused by blunt chest trauma, which can lead to leaflet laceration, rupture of one or more primary chordae, or the tip of the papillary muscle.^6^ Anatomically, the RV is adjacent to the sternum, and it is vulnerable to penetrating injuries of the chest. Theoretically, the disease starts with a rupture of a primary chord during the initial trauma and progresses thereafter by subsequent rupture of adjacent chords due to tension. This is probably related to the thin anatomy of the TV chordae as compared to the mitral chordae. Progressive TR leads to the development of symptoms thereafter.

Echocardiography is the gold standard to evaluate the TV and can be performed after initial patient stabilization.^7^ Sometimes, congenital anatomical conditions may coexist, which further aggravate tension on the involved leaflet. An anterior leaflet cleft was present in our patient complicating the pathology. In the early phase, the patients may be asymptomatic. Symptoms develop as a result of right ventricular dilatation related to volume overload, with subsequent right ventricular dysfunction. Early diagnosis is important for timely treatment to minimize the consequences of right ventricular failure, such as venous congestion and secondary organ dysfunction.^8^

The treatment is mainly surgical by valve repair in the majority of cases, especially in symptomatic patients or those with echocardiographic signs of RV dysfunction. The principle of repair is based on the replacement of ruptured primary chordae by artificial chords.^9^ Concomitant leaflet pathology, such as in our case, should be addressed during valve repair. The TV repair has been associated with high immediate success rates because the condition usually affects young patients with good tissue quality. In cases with severe alterations of the valvular apparatus or presence of subendocardial haematoma, the clover technique followed by annuloplasty or even TV replacement may be necessary.

## Supplementary Material

ytae676_Supplementary_Data

## Data Availability

The data underlying this article are available in the article and in its online Supplementary material.

## Lead author biography

Gianpiero Buttiglione is currently at fourth year of the residency programme in cardiac surgery of University of Milan. He started his training at IRCCS Policlinico San Donato, Milan. Now, he is attending at the Department of Cardiac Surgery, Medical University of Innsbruck. Furthermore, he is a junior representative of the National Council of the Italian Society for Cardiac Surgery in the 2-year period 2023–24.

## Supplementary material

**Consent:** The authors confirm that written consent for the submission and publication of this case report, including images and associated text, has been obtained from the patient, following the COPE guidelines.

**Funding:** This research received no specific grant from any funding agency in the public, commercial, or not-for-profit sectors.
